# Both consolidation and maintenance treatment improve outcomes in primary central nervous system lymphoma: real-world evidence from a tertiary medical center

**DOI:** 10.7150/jca.107661

**Published:** 2025-02-28

**Authors:** Chieh-Lung Cheng, Chang-Tsu Yuan, Wei-Quan Fang, Po-Hao Huang, Hsin-An Hou, Cheng-Hong Tsai, Ming Yao, Wen-Chien Chou, Hwei-Fang Tien

**Affiliations:** 1 Division of Hematology, Department of Internal Medicine, National Taiwan University Hospital, Taipei, Taiwan.; 2 Department of Pathology, National Taiwan University Cancer Center, Taipei, Taiwan.; 3 Division of New Drug, Center for Drug Evaluation, Taipei, Taiwan.; 4 Division of Neurosurgery, Department of Surgery, National Taiwan University Hospital, Taipei, Taiwan.; 5 Department of Internal Medicine, Far-Eastern Memorial Hospital, New Taipei City, Taiwan.

**Keywords:** Primary central nervous system lymphoma, consolidation treatment, maintenance treatment, real-world, prognosis

## Abstract

**Background:** Intensive consolidation treatment following high-dose methotrexate (HDMTX)-based chemotherapy is recommended for fit patients with primary central nervous system lymphoma (PCNSL). Otherwise, HDMTX maintenance might be a useful alternative to consolidation approach in certain circumstances. However, the real-world evidence supporting the beneficial role of consolidation treatment or HDMTX maintenance in PCNSL is limited.

**Methods:** We retrospectively analyzed the clinical efficacy and survival impact of consolidation treatment or HDMTX maintenance on patients with PCNSL treated with HDMTX-based induction chemotherapy.

**Results:** A total of 109 patients were evaluated, with a median age at diagnosis being 63 years. Among them, 69 received induction therapy with HDMTX monotherapy and 40 with HDMTX-based polychemotherapies. In total, 67 (61.5%) patients responded to treatment, of whom 56 (51.4%) had complete response. After a 58.9-month median follow-up, overall survival (OS) at 2 and 5 years was 69% and 45%, respectively. The types of induction regimen or frontline rituximab had no survival impact (*P* = 0.364 and 0.328, respectively). Among the 67 responding patients, 51 received the consolidation/maintenance therapy. Compared to the patients without consolidation/maintenance, those being treated had lower relapse/PD rates (2-year cumulative incidence of relapse/PD, 39.5% vs. 63.6%, *P* <0.001) and a significantly better OS (5-year survival rate, 63.8% vs. 27.2%, *P* = 0.016). Multivariate analysis revealed consolidation/maintenance treatment strikingly reduced mortality risk. Notably, HDMTX maintenance had similar efficacy comparable to consolidative whole-brain radiotherapy. Moreover, consolidation treatment was conducive to prolonging remission duration in the later-line settings of patients who responded to subsequent salvage therapies.

**Conclusion**: This real-world evidence provides clear insight that consolidation/maintenance treatment could prolong OS in PCNSL, emphasizing its critical and indispensable role in treating PCNSL.

## Introduction

Primary central nervous system lymphomas (PCNSLs) are mainly diffuse large B-cell lymphomas (DLBCLs) restricted to the central nervous system (CNS). They typically involve the brain, cerebrospinal fluid (CSF), eyes, and in rare cases, the spinal cord without concomitant systemic disease. PCNSL is now classified as a subtype under a new umbrella term, primary large B-cell lymphoma of immune-privileged sites, by the 2022 World Health Organization classification 1. PCNSL is a rare and aggressive neoplasm, with a median age of 67 years at diagnosis in immunocompetent patients 2. In general, PCNSL has favorable response to both chemotherapy and radiotherapy, but patients with PCNSL frequently experience inferior clinical outcomes when comparing to those with DLBCLs occurring outside of the CNS because of their impaired general condition, poor performance status, and high recurrence rate.

Given the challenges posed by the characteristics of PCNSL, particularly high rates of relapse, consolidation treatment following high-dose methotrexate (HDMTX)-based induction chemotherapy has emerging as an essential component of the overall therapeutic strategy for fit patients with PCNSL 3, 4. The aim of consolidation treatment is to eradicate occult neoplastic cells that may be resistant to the induction therapy, further reducing the risk of relapse and improving survival. Several different consolidative strategies are available for use in clinical practice, and they are guided by the ability to overcome the blood-brain-barrier, the toxicity profiles, and the fitness of the patients. However, the standard consolidation therapy has not yet been defined at the present time.

Generally, there are three modalities of consolidation treatment, including whole-brain radiation therapy (WBRT), nonmyeloablative chemotherapy, and autologous stem cell transplantation (ASCT) using thiotepa-containing conditioning regimens 5-7. Although WBRT at a dose ranging from 36 to 40 Gy was the most used consolidation option for decades, it is being abandoned because of the fear of radiation-induced, late-delayed neurotoxicity 8. Conversely, some studies showed that reduced-dose WBRT (23.4 Gy in 13 fractions) has the benefit of reducing relapse rate and apparently preserving neurological functions in patients aged <60 years with complete response (CR) following induction chemotherapy 9, 10. The efficacy and safety of reduced-dose WBRT are currently being evaluated in a randomized phase 2 trial, with promising preliminary data reported as a meeting abstract 11. Nonmyeloablative consolidative chemotherapy comprising high doses of cytarabine and etoposide showed favorable therapeutic effect but was accompanied by a major obstacle of high risk of hematological and infectious toxicities 12, 13. Moreover, ASCT allows the penetration of high-dose, non-cross-resistant drugs into the CNS, further increasing the possibility of elimination of residual lymphoma cells. Several prospective single-arm or randomized phase 2 studies demonstrated the feasibility and efficacy of thiotepa-containing regimens followed by ASCT as a consolidation option in patients with PCNSL 14-17. Furthermore, a recent international randomized phase 3 trial comparing thiotepa-based ASCT versus non-myeloablative chemoimmunotherapy as the consolidation therapy reported a longer progression-free survival (PFS) in transplanted patients (3-year PFS, 79% vs. 53%) 18. Nevertheless, patient tolerability and treatment-related toxicity remains a major concern in ASCT. Notably, several studies also reported the beneficial role of HDMTX maintenance in patients with PCNSL who have response to the induction therapy 19-21, especially for those who are not eligible for ASCT 22, implying that HDMTX maintenance might be a useful alternative to consolidation treatment in certain circumstances.

Up to now, the benefit of consolidation or maintenance treatment in patients with PCNSL is mainly supported by the non-randomized studies. Only one phase 3, randomized trial examined the risk-benefit issues provided by consolidative WBRT 23, 24. Nevertheless, its serious protocol violations and methodologic flaws preclude the drawing of definite conclusion 25. Additionally, there is no randomized trial proving that HDMTX maintenance is of benefit to PCNSL. Consequently, it is worthy to explore whether patients with PCNSL in real-world settings would benefit from consolidation or maintenance treatment. In this study, we investigated the prognostic impacts provided by consolidation or maintenance treatment in a real-world PCNSL cohort.

## Materials and Methods

### Patient population and Clinical data collection

From January 2000 to December 2022, patients with newly diagnosed PCNSL (CD20+, DLBCL morphology) who received HDMTX-based induction therapy at National Taiwan University Hospital were considered for enrollment. Patients were excluded if they had concomitant systemic lymphoma, a concurrent human immunodeficiency virus infection, or if they had received WBRT as their initial treatment. All patients underwent brain magnetic resonance imaging (MRI) and whole-body computed tomography (CT) with contrast or positron emission tomography/CT to clarify the disease extent. Diagnostic CSF analysis to evaluate leptomeningeal disease was only performed for patients without imminent risk of brain herniation.

A retrospective chart review was performed to collect data on clinical characteristics, treatment responses, relapse events, treatment-related toxicities, and survival outcomes. Treatment-related toxicities were evaluated in accordance with the National Cancer Institute Common Terminology Criteria for Adverse Events Version 5. The International Extranodal Lymphoma Study Group (IELSG) and Memorial Sloan Kettering Cancer Center (MSKCC) prognostic scores were obtained as the previously reported 26, 27.

### Induction, consolidation, and maintenance treatment

All enrolled patients had received at least one cycle of induction chemotherapy with HDMTX-based mono- or polychemotherapies. For HDMTX monotherapy, it was administered at a target dose of 8 g/m^2^ over 6 hours under a biweekly schedule. Dose reduction of MTX was acceptable but at doses of at least 3.5 g/m^2^ per cycle. For HDMTX-based polychemotherapy, MTX was administered at doses of at least 1.5 g/m^2^ per cycle. Intravitreal injection of MTX was applied to patients with ocular involvement. Those with concomitant leptomeningeal diseases also received concurrent intrathecal chemotherapy consisting of MTX, cytarabine, and hydrolcortisone.

Consolidation or maintenance treatment was defined as a therapy that is delivered for a patient achieving a CR or partial response (PR) after the induction chemotherapy. The consolidation options included consolidative WBRT, ASCT, or nonmyeloablative chemotherapy. Reduced-dose WBRT was also regarded as one of the consolidation options. The maintenance therapy in the present study only included monthly HDMTX. The dose of monthly HDMTX was the same as that used in the induction protocol and the duration of monthly HDMTX therapy would be up to 1 year whenever possible.

### Evaluation of treatment response

The assessment of therapeutic response was assessed using contrast-enhanced MRI in all patients at the end of frontline treatment. CR, unconfirmed CR (CRu), PR, progressive disease (PD), and stable disease (SD) are determined using the International Primary CNS Lymphoma Collaborative Group criteria 28. The ORR is defined as the proportion of patients whose best response was either CR or PR. Relapsed disease means re-appearance of any new lesion after achieving CR/CRu.

### Statistical analysis

We used the chi-square test or Fisher's exact test to compare categorical data. A Mann-Whitney U test was used to compare the medians of continuous variables. Overall survival (OS) was defined as the time from the date of diagnosis to either death from any cause or the last date of follow-up. PFS was measured from the date of diagnosis until the occurrence of the earliest event among the following: 1) relapse or PD, 2) death, or 3) the last date of follow-up. Importantly, to assess the benefits of consolidation/maintenance treatment among the responding patients, OS was measured from the date they achieved CR or PR after induction treatment to the date of death from any cause. Similarly, PFS for these patients was measured from the date of achieving CR or PR after induction treatment to the time of progression, relapse, death from any cause, or the last date of follow-up. Cumulative incidence curves were determined for relapse or PD events, and Gray's test was used to examine the significance of between-group differences. The Kaplan-Meier method was used to calculate the survival curves, and a log-rank test was used to identify significant between-group differences. Notably, landmark (LM) analyses were performed to correct the immortal time bias in the consolidation/maintenance group. Hazard ratios (HRs) and 95% confidence intervals (CIs) were estimated using univariate and multivariate Cox proportional hazard regression models to determine the independent risk factors associated with survival. A two-sided *P* value <0.05 was considered statistically significant.

## Results

### Demographics

A total of 109 patients were evaluated, with a median age at diagnosis being 63 years (range, 24-86 years) and 55 (50.5%) being women. Among them, 24 underwent surgical resection, 76 stereotactic biopsy, and 9 CSF analysis as the diagnostic approach for PCNSL. Additionally, 42 patients had unifocal brain lesion, 62 multifocal brain lesion, and 5 primary leptomeningeal disease. Furthermore, 87 (79.8%) patients had an Eastern Cooperative Oncology Group (ECOG) performance status of ≥2, 43 (39.4%) had the Karnofsky Performance Scale less than 70, 28 (25.7%) had a high IELSG prognostic score, and 39 (35.8%) belonged to the MSKCC high-risk group. The detailed clinical features of these 109 patients were shown in Table 1.

### Induction chemotherapy

Among the cohort, 69 (63.3%) patients received HDMTX monotherapy as the induction chemotherapy. The median number of HDMTX cycles was 5 (range, 1-9). Additionally, 51 of them (73.9%) received rituximab as a part of induction therapy (Supplementary Table S1). By contrast, HDMTX-based polychemotherapies were delivered to 40 (36.7%) patients, of whom 26 received carmustine (BCNU), vincristine, methotrexate, etoposide, and methylprednisolone 29, 6 HDMTX with temozolomide, 3 HDMTX with BCNU, 3 HDMTX, BCNU and cytarabine, and 2 HDMTX with high-dose cytarabine (HDAC). The median number of treatment cycles was 4 (range, 1-9). Notably, the percentage of frontline rituximab used in this group was 40%, which was significantly lower than that in the HDMTX-monotherapy group (*P* <0.001, Supplementary Table S1).

### Treatment response

Sixty-seven patients had response to the induction treatment. The ORR and CR/CRu rate was 61.5% and 51.4%, respectively, whereas the SD/PD rate was 30.2%. The addition of frontline rituximab did not improve treatment response. Notably, the ORR, CR/CRu rates, and SD/PD rates had no significant differences between the patients with HDMTX monotherapy and those receiving HDMTX-based polychemotherapies (Supplementary Table S1).

### Consolidation/maintenance treatment

Of the 67 responding patients, 51 received the subsequent consolidation or maintenance treatment, of which 19 were consolidative WBRT (including 3 reduced-dose WBRT), 4 nonmyeloablative chemotherapy, 6 ASCT, and 22 monthly HDMTX maintenance. The median consolidative whole-brain dose was 40 Gy (range, 23.4-45 Gy). The nonmyeloablative consolidative chemotherapies were all HDAC-containing regimens administered for at least two cycles (range, 2-4). Additionally, the conditioning regimens delivered to six patients with consolidative ASCT all contained thiotepa, of which 3 were thiotepa/BCNU, 1 thiotepa/BCNU/etoposide, 1 thiotepa/busulfan, and 1 thiotepa/busulfan/cyclophosphamide. Moreover, the median number of monthly HDMTX cycles was 6 (range, 2-12). Compared to the patients in the HDMTX-based-polychemotherapy group, those in the HDMTX-monotherapy group more frequently received monthly HDMTX maintenance (61.1% vs. 0%,* P* <0.001). By contrast, the patients in the HDMTX-based-polychemotherapy group more frequently received consolidative WBRT as compared to those in the HDMTX-monotherapy group. (80% vs. 19.4%, *P* <0.001).

### Survival

A total of 53 patients died at the time of the last follow-up. The most common two causes of death were relapsed PCNSL (40/53, 75.5%) and neurotoxicity (5/53, 9.4%). After a median follow-up time of 58.9 months, the median OS was 44 months. OS rate at 2 and 5 years was 69% and 45%, respectively (Supplementary Fig. S1A). Eighty-three patients experienced PFS events, of which 73 (87.9%) were caused by PCNSL. The median PFS was 13.3 months (95% CI, 9.4 to 21.7 months), and the 2-year and 5-year cumulative PFS rates were 35.1% and 15.9%, respectively (Supplementary Fig. S1B).

The univariate analysis of clinical characteristics associated with OS is presented in Supplementary Table S2. OS rate at 2 and 5 years was only 49% and 21%, respectively, for the patients with high-risk IELSG scores. The patients in the high-risk MSKCC group also had the worst outcomes (2-year and 5-year OS rates of 47.7% and 25.5%, respectively). Furthermore, multifocal/diffuse brain lesion, pretreatment serum albumin level less than 3.5 g/mL, and no response to induction chemotherapy had unfavorable impacts on survival. Notably, the diagnostic strategy for PCNSL, the type of induction chemotherapy, or the addition of rituximab to the induction therapy had no prognostic impact. Multivariate analysis of variables significantly associated with survival in univariate analysis identified low pretreatment serum albumin level, no response to induction chemotherapy, and high-risk MSKCC group as the independent unfavorable prognostic factors for OS (Supplementary Table S2).

### Clinical features of patients with or without consolidation/maintenance treatment

The clinical characteristics of 67 responding patients with or without consolidation/maintenance treatment are presented in Supplementary Table S3. Most clinical features were similar between the two groups. The patients with PR to the induction therapy all received the subsequent consolidation/maintenance treatment. Notably, the patients without consolidation/maintenance treatment more frequently underwent surgical resection as the diagnostic strategy and received the HDMTX-based polychemotherapies as the induction treatment than the patients being treated.

### Prognostic impact of consolidation/maintenance treatment

**Figure 1:**
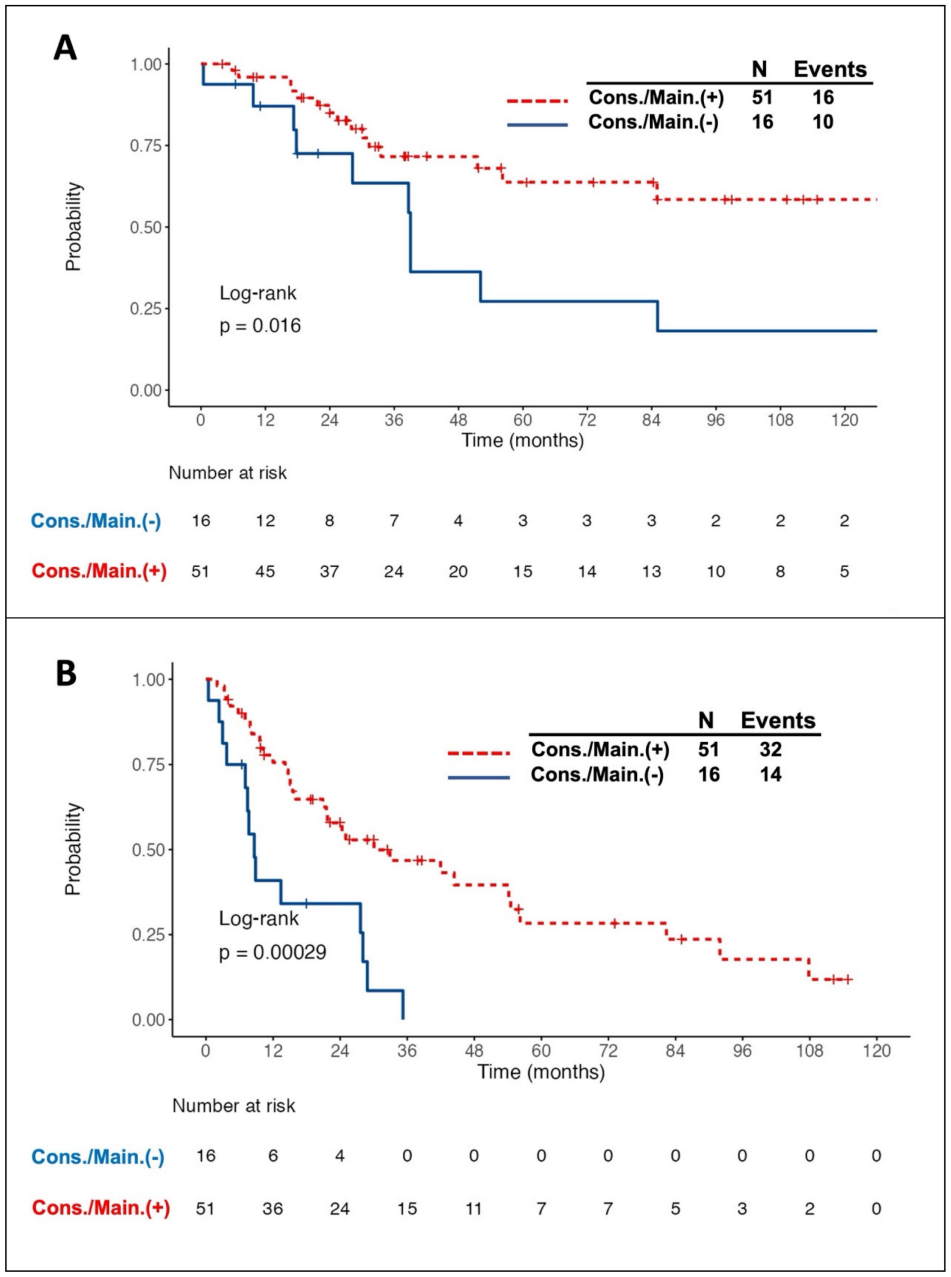
**Kaplan-Meier survival curves in patients with primary central nervous system lymphoma who responded to the induction chemotherapy, stratified by the adoption of consolidation/maintenance (cons./main.) treatment or not.** Patients with cons./main. treatment had a significantly better overall survival (OS) (A) and longer progression-free survival (PFS) (B) than those without treatment. Notably, OS and PFS were calculated from the time of achieving a complete or partial response after induction treatment.

**Figure 2:**
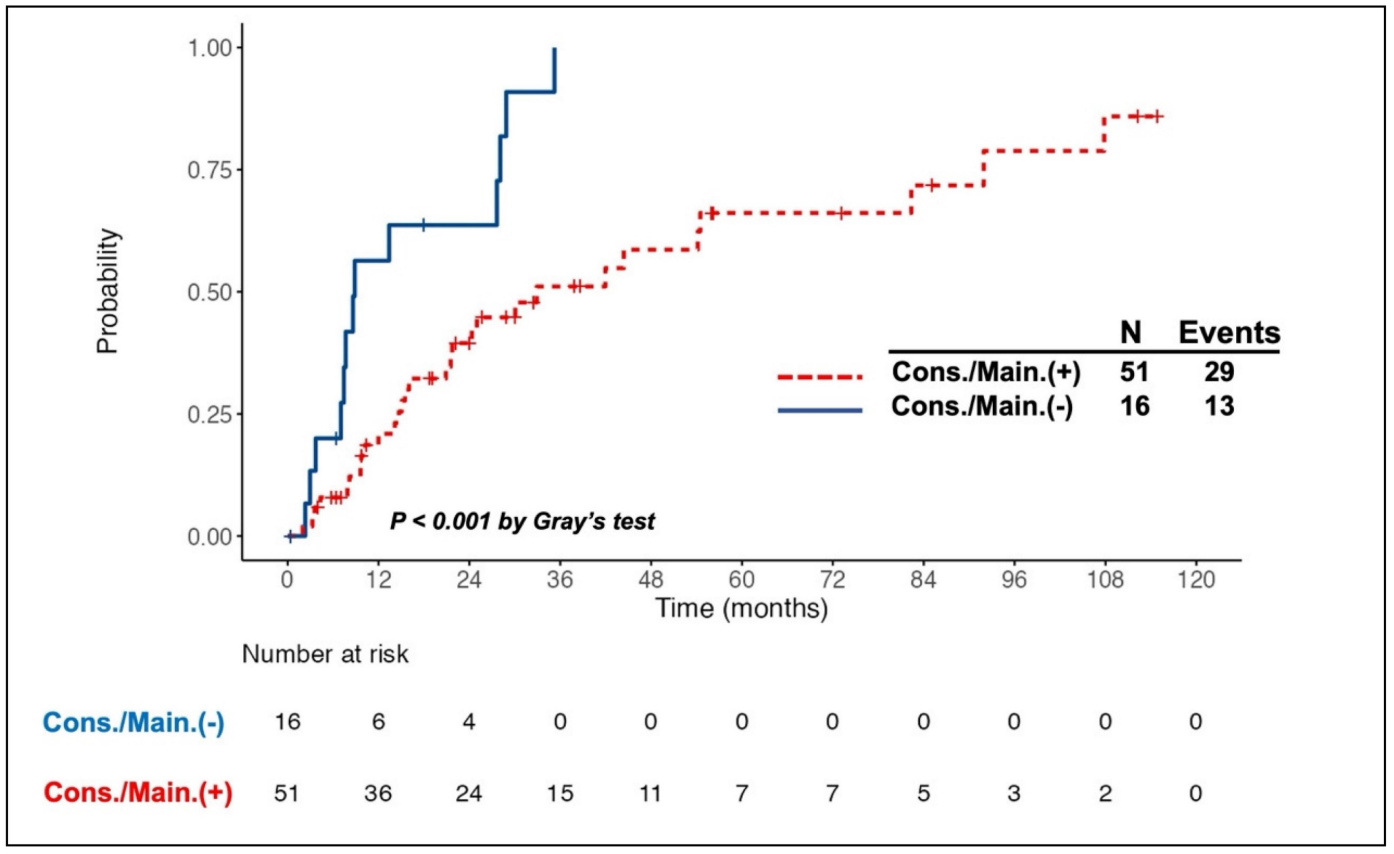
**Cumulative incidence of relapse/progressive disease (PD) in patients with primary central nervous system lymphoma who responded to the induction chemotherapy, stratified by the adoption of consolidation/maintenance (cons./main.) treatment or not.** A higher cumulative incidence rate (CIR) of relapse/PD was observed in patients without cons./main. treatment than in those being treated. The CIR of relapse/PD at 2 years after achieving a complete or partial response was 39.5% and 63.6% in patients with and without cons./main. treatment, respectively.

Intriguingly, all 11 patients with PR to induction therapy achieved CR/CRu after the consolidation/maintenance treatment. Among the total of 67 responding patients, 26 died, of whom 10 did not receive the consolidation/maintenance treatment. Compared to the patients without consolidation/maintenance treatment, those being treated had a better 5-year OS rate (63.8% vs. 27.2%, log-rank *P* = 0.016, Fig. 1A) and longer median PFS (30.1 months vs. 8.6 months, log-rank *P* <0.001, Fig. 1B). The survival benefit of consolidation/maintenance treatment was also confirmed at LM 3 months (*P* = 0.044) and LM 6 months (*P* = 0.03), respectively. Moreover, 42 patients experienced relapsed/PD events, of whom 13 were in the unconsolidated/unmaintained group. The cumulative incidence rate of relapse/PD at 2 years was 63.6% (95% CI, 36.6% to 90.7%) in the unconsolidated/unmaintained group and 39.5% (95% CI, 24.9% to 54%) in the treatment group (*P* <0.001, Fig. 2).

Multivariate analysis revealed that consolidation/maintenance treatment is an independent favorable factor for survival (HR, 0.41; 95% CI, 0.18-0.97; *P* = 0.042, Table 2) in the 67 responding patients, irrespective of types of induction therapy and the MSKCC prognostic model.

Notably, of the 33 patients with SD/PD to the induction therapy, 32 received the subsequent salvage therapies (15 BCNU-containing regimen, 4 HDAC, 6 radiotherapy followed by systemic chemotherapy, and 7 radiotherapy alone). Among them, 18 achieved responses. Of these 18 responders, 8 also received further consolidation treatment (1 WBRT, 5 nonmyeloablative chemotherapy, and 2 ASCT). These 8 consolidated patients still had a trend of better prognosis than 10 others without consolidation treatment (2-year OS rate, 85.7% vs. 36%, *P* = 0.17; 2-year PFS rate, 71.4% vs. 30%, *P* = 0.083, Supplementary Fig. S2).

### Survival benefit of consolidation/maintenance treatment in different types of induction chemotherapy

Of the 67 responding patients, 42 received HDMTX monotherapy and 25 HDMTX-based polychemotherapies as the induction therapy, respectively. In the HDMTX-monotherapy group, the patients with consolidation/maintenance treatment had a trend of higher 5-year OS rate as compared to those without treatment (61% vs. 20%, *P* = 0.074, Supplementary Fig. S3A). Similarly, in the HDMTX-based-polychemotherapy group, the patients with consolidation/maintenance treatment had a trend of better clinical outcome as compared to the untreated patients (5-year OS rate, 75.5% vs. 33.3%, *P* = 0.11, Supplementary Fig. S3B).

### Outcome and treatment-related adverse effects of patients with different consolidation/maintenance modalities

**Figure 3:**
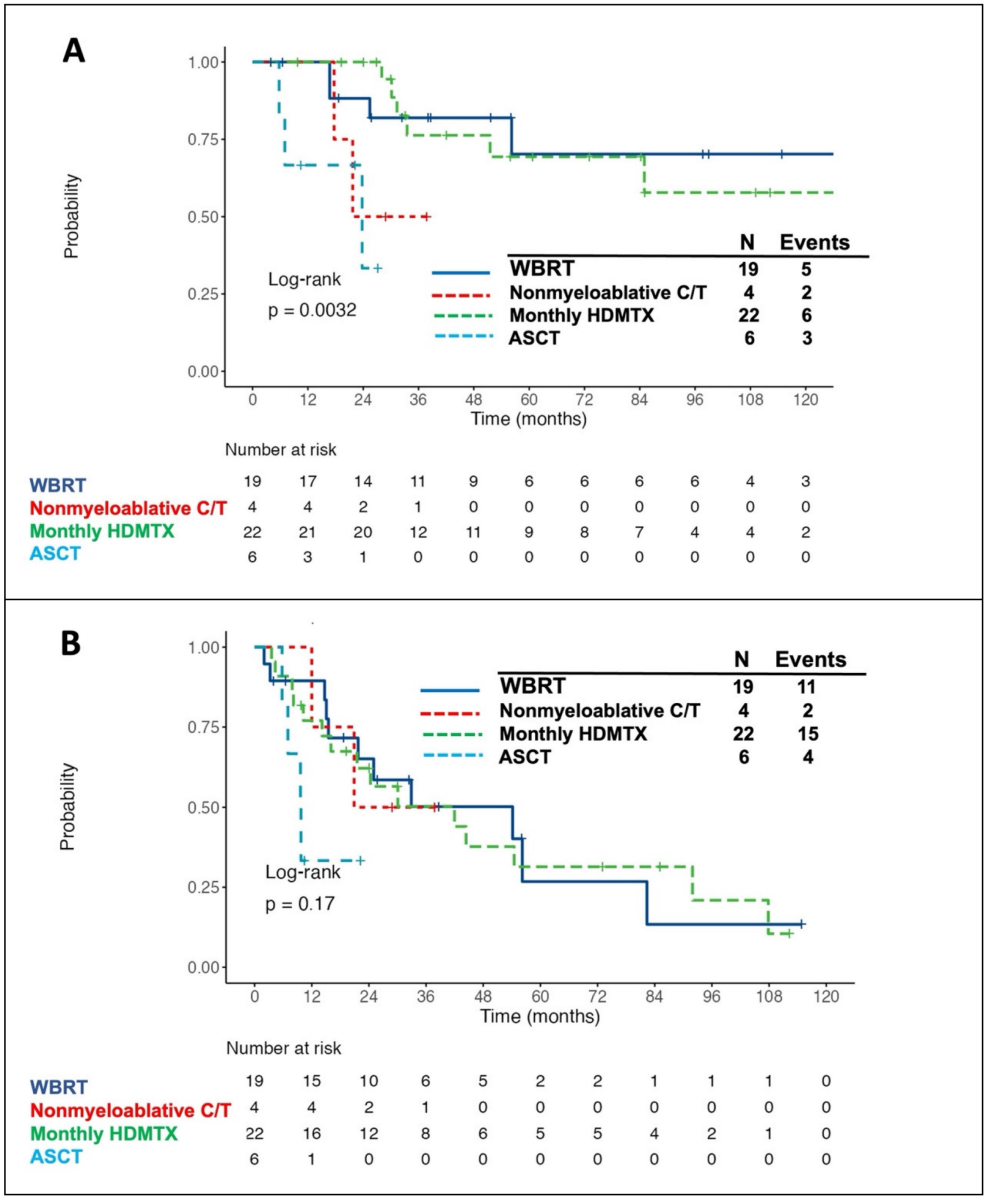
**Kaplan-Meier survival curves in patients with primary central nervous system lymphoma, stratified by the types of consolidation/maintenance treatment.** The patients with consolidative WBRT or with monthly HDMTX maintenance had the best 5-year overall survival (OS) (A, 70.2% for consolidative WBRT and 69.4% for monthly HDMTX maintenance) and 3-year progression-free survival (PFS) (B, 50.2% for consolidative WBRT and 50.3% for monthly HDMTX maintenance) rates. Notably, OS and PFS were calculated from the time of achieving a complete or partial response after induction treatment. Abbreviations: WBRT, whole-brain radiotherapy; HDMTX, high-dose methotrexate; C/T, chemotherapy; ASCT, autologous stem cell transplantation.

The survival curves for patients receiving different consolidation/maintenance options were depicted in Fig. 3. Of the 51 patients with consolidation/maintenance treatment, 16 died. The most common cause of death was PD due to lymphoma (12/16, 75%), followed by cytomegalovirus (CMV) pneumonia (2/16, 12.5%) and neurotoxicity (2/16, 12.5%). Of note, patients with consolidative WBRT or with monthly HDMTX maintenance had the best 5-year OS rate (Fig. 3A) and 3-year PFS rate (Fig. 3B). Of the four patients receiving nonmyeloablative consolidative chemotherapy, 2 died-both from PD of lymphoma. Three of six patients under the consolidative ASCT setting died-two from CMV pneumonia post transplantation and one from recurrent disease. Five out of 19 (26.3%) patients with consolidative WBRT developed delayed neurotoxicity, and two of them died-one aged 54 years with the standard whole-brain dose of 45 Gy and the other aged 84 years with the reduced dose of 23.4 Gy. Furthermore, all but five (17/22, 77.3%) patients with monthly HDMTX maintenance developed hepatotoxicity, of which ten was grade 1, five was grade 2, and two was grade 3 toxicity. Four (18.2%) patients developed HDMTX-related nephrotoxicity, among whom two had grade 1 and the other two had grade 2 toxicity. Notably, both hepatotoxicity and nephrotoxicity were reversible without late sequelae. Concerning the hematologic toxicities, three (13.6%) patients developed grade 3/4 neutropenia. Besides, grade 1/2 thrombocytopenia was observed in three (13.6%) patients, and no grade 3/4 anemia or thrombocytopenia was recorded. Overall, there was no toxic death of HDMTX maintenance.

## Discussion

Consolidation or maintenance treatment plays a pivotal role in the management of PCNSL, with an intent to eliminate residual disease, reduce the risk of relapse, and improve survival. Currently, few randomized studies directly compared the outcomes of patients with or without consolidation/maintenance treatment, whereas the benefit of this treatment approach is mainly based on the non-randomized clinical trials 30. This study investigated the prognostic implications of consolidation or maintenance treatment in a real-world PCNSL cohort, showing that this treatment approach remarkably reduced the disease relapse or progression and improved survival. Notably, we demonstrated that monthly HDMTX maintenance yielded similar OS and PFS rates comparable to those involving consolidative WBRT. Moreover, the benefit of consolidation treatment could be reproduced in patients with SD/PD who had response to subsequent salvage therapies. This real-world evidence corroborates the efficacy of consolidation or maintenance treatment derived from the clinical trials, further strengthening the importance of adoption of this treatment approach for patients with PCNSL whenever possible.

Owing to the radiosensitive nature of PCNSL, WBRT has been regarded as a standard treatment in the past and was routinely applied to patients with PCNSL in clinical practice. Furthermore, the addition of consolidative WBRT following HDMTX-based induction therapy showed a 2-fold increase of median survival as compared to the standard WBRT alone, with a median OS of 30-60 months and a 5-year survival rate of 30-50% 31-33. Nevertheless, this combined modality therapy comes at a cost of delayed neurotoxicity, especially for patients aged 60 years and older,^8^ which leads to an attempt to reduce the neurotoxicity by consolidating with reduced-dose WBRT or with hippocampal-avoidance WBRT 11, 34, 35. In our cohort, the patients who received the consolidative WBRT in the frontline setting also had a higher 5-year OS rate as compared to those without consolidation (70.2% vs. 27.2%), further supporting the treatment efficacy of consolidative WBRT. However, two patients died of delayed neurotoxicity-one with the standard whole-brain dose and the other with the reduced dose. The potential benefit of reduced-dose WBRT to reduce the risk of delayed neurotoxicity and preserve treatment efficacy could not be evaluated in our study due to the limitation on number of cases.

Except for reduced-dose WBRT, alternative strategies have been utilized to improve disease outcomes and avoid the adverse effect of delayed neurotoxicity incurred by WBRT. These include nonmyeloablative chemotherapy or high-dose chemotherapy followed by ASCT as the consolidation treatment. Two prospective, randomized phase 2 studies compared the outcomes of patients with PCNSL who received Thiotepa-based ASCT consolidation versus standard WBRT consolidation, revealing that these two modalities had similar efficacy, whereas WBRT was associated with a prominent decline in neurocognitive function when compared to ASCT consolidation 16, 17. In our cohort, three of the six patients undergoing thiotepa-based ASCT consolidation died. The 2-year OS rate from consolidation in transplanted patients was only 33.3%, which was obviously inferior to that reported in the clinical trials. Notably, two out of the three deaths were related to CMV pneumonia post transplantation. It has been reported that thiotepa-based conditioning regimen is associated with a risk of DNA viral reactivation and infection in patients with CNS lymphoma 36. This might partially explain the unfavorable prognosis of patients undergoing ASCT consolidation in our cohort. Regarding the nonmyeloablative chemotherapy, consolidation with high-dose etoposide plus cytarabine following the induction therapy yielded favorable PFS and OS, but at the expense of toxic adverse effects 12, 13. One recent randomized phase 2 trial also showed that both ASCT and nonmyeloablative consolidation yielded encouraging efficacy 37. However, the largest randomized phase III study reported the superior benefit of consolidative ASCT over non-myeloablative chemotherapy on newly diagnosed PCNSL patients, implying that fit patients should properly be approached to ASCT 18. Owing to the limited number of cases, the efficacy of nonmyeloablative consolidative chemotherapy was unclear in our study.

Apart from the three modalities generally utilized as the consolidation treatment, several studies had reported that maintenance therapy with single-agent HDMTX might be a useful alternative. Yoon *et al.* showed a modest 2-year PFS rate of 24% in patients receiving induction therapy with 8 g/m^2^ of HDMTX monotherapy followed by maintenance therapy with 3.5 g/m^2^ of HDMTX 20. A retrospective study consisting of 52 patients with PCNSL demonstrated that continued maintenance HDMTX monotherapy at a dose of 3.5 g/m^2^ had a high 3-year OS rate of 74.9% 21. Furthermore, comparable outcomes were observed in PCNSL between a post-induction consolidation approach with ASCT and maintenance HDMTX 22. In the present study, maintenance therapy with monthly HDMTX had a high 5-year OS rate of 69.4% and 3-year PFS rate of 50.3%. Besides, it was well tolerated with acceptable toxic profiles. Our results suggested that monthly HDMTX is a good alternative to consolidation treatment and might be a reasonable choice for patients with PCNSL who are unfit for consolidative WBRT or other intensive consolidation therapies.

In addition to HDMTX maintenance, several studies or case reports showed that other chemotherapies (e.g., temozolomide or procarbazine) or targeted agents (e.g., lenalidomide or inhibitors targeting Bruton's tyrosine kinase), might have a role as maintenance therapy in PCNSL, particularly in the frail and elderly patients who cannot tolerate intensive consolidation treatment 38-43. The rationale of maintenance therapy is to maintain cancer dormancy and delay relapses, further creating opportunities for long-term disease remission 44. Although a phase 3 study failed to demonstrate the advantage of maintenance therapy with temozolomide 45, a recent prospective phase 2 trial evaluating ibrutinib maintenance in older patients with PCNSL showed good tolerability with high 2-year PFS and OS rates of 72.6% and 89%, respectively 46. Further randomized studies are required to investigate whether maintenance therapy with these novel targeted agents are optional alternatives to conventional consolidation approach in the elderly.

Most studies investigate the role of consolidation treatment in the frontline setting, whereas the benefit of consolidation treatment in the second or subsequent lines of treatment is mainly based on the results of high-dose chemotherapy followed by ASCT in patients with relapsed or refractory PCNSL, with 2-year PFS and OS rates of 46%-58% and 56.4%-69%, respectively 47-49. Although the number of cases was limited, our study also showed that the patients in the second-line setting who responded to the salvage therapies might benefit from consolidation treatment. The majority of consolidative strategies utilized under this circumstance in the present study were nonmyeloablative chemotherapy, suggesting that the consolidation approach, including but not limited to ASCT, might be conducive to prolonging remission duration in later-line settings of patients with PCNSL.

The main limitation of our study is the inherent obstacle of existing potential biases or confounders due to the retrospective nature spanning a long period. Additionally, the radiation-induced, late-delayed neurotoxicity and cognitive deficits caused by WBRT could not be prospectively and accurately evaluated by neuropsychological testing and neuroimaging. Furthermore, the number of patients receiving nonmyeloablative consolidative chemotherapy or ASCT consolidation was limited, thus we could not conduct an individual assessment of the survival impact of each of the two consolidative strategies on patients with PCNSL through this study. The cost of thiotepa as part of PCNSL conditioning regimens is not covered by our National Health Insurance Reimbursement, which might partially explain the limited number of patients undergoing ASCT consolidation. Theoretically, prospective, randomized studies comparing the outcomes between patients with or without consolidation/maintenance treatment are warranted to explore the true survival impact provided by different options of consolidation/maintenance treatment. Currently, it is hard to conduct these trials due to the ethical issues.

In conclusion, this real-world study provides supportive evidence that consolidation or maintenance treatment could reduce the risk of relapse and improve survival in patients with PCNSL. Intriguingly, we demonstrated that the efficacy of maintenance therapy with monthly HDMTX was like that involving consolidative WBRT, indicating that HDMTX maintenance might be an optional alternative to conventional consolidation treatment. Of interest, not only is consolidation treatment beneficial in the frontline setting, but it also provides additional supportive evidence in the later-line treatment, underscoring that consolidation/maintenance treatment is of the utmost importance in the overall therapeutic strategy of PCNSL.

## Supplementary Material

Supplementary figures and tables.

## Figures and Tables

**Table 1:** Patient characteristics

Patietnt demographics	N = 109
Median age at diagnosis (range)	63 (24-86)
Sex†	
Male	54 (49.5)
Female	55 (50.5)
**Diagnostic strategy**†	
Surgical resection	24 (22)
Stereotactic biopsy	76 (69.7)
CSF analysis	9 (8.3)
**Clinical features**†	
Disease involvement	
Unifocal	42 (38.5)
Multifocal	62 (56.9)
Primary leptomeningeal	5 (4.6)
Meningeal involvement	
Yes	30 (27.5)
No	65 (59.7)
Unknown	14 (12.8)
Ocular involvement	
Yes	14 (12.8)
No	48 (44.1)
Unknown	47 (43.1)
ECOG Performance status ≥2	87 (79.8)
KPS <70	43 (39.4)
Serum LDH >1x ULN	24 (22)
Serum albumin <3.5 g/dL#	27/89 (30.3)
**IELSG risk group**†	
Low	14 (12.8)
Intermediate	61 (56)
High	28 (25.7)
Unknown	6 (5.5)
**MSKCC risk group**†	
Low	16 (14.7)
Intermediate	54 (49.5)
High	39 (35.8)
**Induction treatment**†	
HDMTX monotherapy	69 (63.3)
HDMTX-based polychemotherapy	40 (36.7)
**Induction treatment with rituximab**†	67 (61.5)
**Treatment response to induction therapy**†	
CR/CRu	56 (51.4)
PR	11 (10.1)
SD/PD	33 (30.2)
Unknown@	9 (8.3)
**Autologous/allogeneic stem cell transplantation**†*	
Frontline consolidation	6 (5.5)
Second-line consolidation	2 (1.8)
Relapse/salvage	4 (3.7)
**WBRT**†	
Frontline consolidation	19 (17.4)
Second-line consolidation	1 (0.9)
Relapse/salvage	31 (28.4)
**Non-myeloablative chemotherapy**	
Frontline consolidation	4 (3.7)
Second-line consolidation	5 (4.6)
**Maintenance HDMTX**	22 (20.2)

† Number of patients (%) # Eighty-nine patients had the pretreatment serum albumin data. @ Including four patients with induction death and five patients who were lost to follow up. * All cases were autologous stem cell transplantation except for one allogeneic stem cell transplantation in the relapse/salvage setting. Abbreviations: CSF, cerebrospinal fluid; ECOG, Eastern Cooperative Oncology Group; KPS, Karnofsky Performance Scale; LDH, lactate dehydrogenase; ULN, upper limit of normal; IELSG, International Extranodal Lymphoma Study Group; MSKCC, Memorial Sloan Kettering Cancer Center; HDMTX, high-dose methotrexate; BOMES, carmustine, vincristine, methotrexate, etoposide, and methylprednisolone; CR, complete response; CRu, unconfirmed complete response; PR, partial response; SD, stable disease; PD, progressive disease; WBRT, whole-brain radiotherapy.

**Table 2:** Univariate and multivariate analyses of risk factors for examining overall survival in the responding patients

	Univariate analysis			Multivariate analysis		
Variables	HR	95% CI	*P* value	HR	95% CI	*P* value
High-risk MSKCC group	2.7	1.23-5.93	0.013	2.34	1.04-5.31	0.041
Induction treatment†	1.13	0.48-2.62	0.781	1.02	0.42-2.49	0.957
Consolidation/maintenance	0.35	0.16-0.78	0.011	0.41	0.18-0.97	0.042

† High-dose methotrexate-based polychemotherapies versus High-dose methotrexate monotherapy. Abbreviations: HR, hazard ratio; CI, confidence interval; MSKCC, Memorial Sloan Kettering Cancer Center.
