# Supplementary material for: Both consolidation and maintenance treatment improve outcomes in primary central nervous system lymphoma: real-world evidence from a tertiary medical center
Source: J Cancer. 2025 Feb 28;16(6):1836–47. doi: 10.7150/jca.107661 (PMC11905409; doi:10.7150/jca.107661)
Supplement: Supplementary file 1 — Supplementary figures and tables. [file jcav16p1836s1.pdf]

Supplementary data

Table S1. Clinical characteristics of 109 patients with primary central nervous system lymphoma

Variables	HDMTX monotherapy (n = 69)	HDMTX-based polychemotherapies (n = 40)	*P* value
**Age at diagnosis, years‡**	64 (39-86)	61 (24-78)	0.061
**Sex†**			0.746
Male	35 (50.7)	19 (47.5)	
Female	34 (49.3)	21 (52.5)	
**Diagnostic strategy†**			0.013
Surgical resection	10 (14.5)	14 (35)	
Stereotactic biopsy/CSF analysis	59 (85.5)	26 (65)	
**Disease involvement†**			0.143
Unifocal	23 (33.3)	19 (47.5)	
Multifocal/primary leptomeningeal	46 (66.7)	21 (52.5)	
**Meningeal involvement†**			0.006
Present	25 (36.2)	5 (12.5)	
Absent	39 (56.5)	26 (65)	
Unknown	5 (7.3)	9 (22.5)	
**Ocular involvement†**			<0.001
Present	11 (15.9)	3 (7.5)	
Absent	38 (55.1)	10 (25)	
Unknown	20 (29)	27 (67.5)	
**ECOG PS†**			0.05
0, 1	18 (26.1)	4 (10)	
≥2	51 (73.9)	36 (90)	
**Serum LDH†**			0.003
Elevated	9 (13)	15 (27.5)	
Normal	60 (87)	25 (62.5)	
**Serum albumin†**			0.966
<3.5 g/dL	17 (24.6)	10 (25)	
≥3.5 g/dL	52 (75.4)	30 (75)	
**High-risk IELSG score†**	19 (27.5)	9 (22.5)	0.562
**High-risk MSKCC group†**	26 (37.7)	13 (32.5)	0.587
**Frontline rituximab†**	51 (73.9)	16 (40)	<0.001
**Treatment response†**			0.192
Overall response	42 (60.9)	25 (62.5)	
CR/CRu	35 (50.7)	21 (52.5)	
PR	7 (10.2)	4 (10)	
SD/PD	24 (34.8)	9 (22.5)	

Not evaluated@	3 (4.3)	6 (15)

‡ Median (range)
† Number of patients (%)
@ Including four patients with induction death and five patients who were lost to follow up.
Abbreviations: HDMTX, high-dose methotrexate; CSF, cerebrospinal fluid; ECOG PS, Eastern Cooperative Oncology Group Performance Status; LDH, lactate dehydrogenase; IELSG, International Extranodal Lymphoma Study Group; MSKCC, Memorial Sloan Kettering Cancer Center; HDMTX, high-dose methotrexate; CR, complete response; CRu, unconfirmed complete response; PR, partial response; SD, stable disease; PD, progressive disease.

Table S2. Univariate and multivariate analyses of risk factors for examing overall survival in patients with PCNSL

	Univariate analysis			Multivariate analysis		
**Variables**	HR	95% CI	*P* value	HR	95% CI	*P* value
Age >60 years	1.36	0.78–2.36	0.282			
Sex (Male vs. Female)	1.45	0.84–2.5	0.186			
Diagnostic strategy@	1.3	0.7–2.41	0.403			
Extent of disease#	1.99	1.08–3.68	0.028*	1.31	0.67–2.58	0.428
Meningeal involvement	1.7	0.94–3.09	0.081			
Ocular involvement	0.63	0.25–1.54	0.306			
ECOG PS (≥2 vs. 0,1)	1.98	0.96–4.09	0.065			
KPS <70	3.92	2.23–6.87	<0.001*			
Serum LDH >1x ULN	5.18	2.75–9.75	<0.001*			
Serum albumin <3.5 g/dL	3.16	1.74–5.75	<0.001*	2.14	1.13–4.04	0.019
High-risk IELSG score	2.14	1.18–3.86	0.012*	1.55	0.83–2.9	0.168
High-risk MSKCC group	3.18	1.84–5.51	<0.001*	2.06	1.15–3.7	0.016
Induction treatment†	1.31	0.73–2.32	0.364			
Frontline rituximab	0.76	0.44–1.31	0.328			
Response to initial therapy‡	3.95	2.27–6.89	<0.001*	3.63	1.96–6.75	< 0.001

@ Surgery resection versus stereotactic biopsy/cerebrospinal fluid analysis.
# Multifocal/primary leptomeningeal disease versus unifocal lesion.
† High-dose methotrexate-based polychemotherapies versus High-dose methotrexate monotherapy.
‡ Stable disease/progressive disease/not evaluable versus complete response/unconfirmed complete response/partial response.
*All potential prognostic factors for overall survival with a *P* value less than 0.05 in the univariate analysis were selected for multivariate analysis, except for those redundant with IELSG or MSKCC prognostic score.
Abbreviations: PCNSL, primary central nervous system lymphoma; HR, hazard ratio; CI, confidence interval; ECOG PS, Eastern Cooperative Oncology Group Performance Status; LDH, lactate dehydrogenase; ULN, upper limit of normal; IELSG, International Extranodal Lymphoma Study Group; MSKCC, Memorial Sloan Kettering Cancer Center.

Table S3. Demographic and clinical characteristics of patients who responded to the induction therapy

Variables	With consolidation/ maintenance (n = 51)	Without consolidation/ maintenance (n = 16)	*P* value
**Age at diagnosis, years‡**	60 (39-84)	63 (24-86)	0.994
**Sex†**			0.392
Male	27 (52.9)	6 (37.5)	
Female	24 (47.1)	10 (62.5)	
**Diagnostic strategy†**			0.029
Surgical resection	7 (13.7)	7 (43.7)	
Stereotactic biopsy/CSF analysis	44 (86.3)	9 (56.3)	
**Disease involvement†**			0.775
Unifocal	22 (43.1)	8 (50)	
Multifocal/primary leptomeningeal	29 (56.9)	8 (50)	
**Meningeal involvement†**			0.291
Present	12 (23.5)	7 (43.7)	
Absent	34 (66.7)	8 (50)	
Unknown	5 (9.8)	1 (6.3)	
**Ocular involvement†**			0.169
Present	8 (15.7)	3 (18.7)	
Absent	26 (51)	4 (25)	
Unknown	17 (33.3)	9 (56.3)	
**ECOG PS†**			0.527
0, 1	15 (39.4)	3 (18.7)	
≥2	36 (70.6)	13 (81.3)	
**Serum LDH†**			0.05
Elevated	3 (5.9)	4 (25)	
Normal	48 (94.1)	12 (75)	
**Serum albumin†**			0.716
<3.5 g/dL	8 (15.7)	3 (18.7)	
≥3.5 g/dL	43 (84.3)	13 (81.3)	
**High-risk IELSG score†**	10 (19.6)	4 (25)	0.73
**High-risk MSKCC group†**	12 (23.5)	7 (43.7)	0.202
**Induction treatment†**			0.036
HDMTX monotherapy	36 (70.6)	6 (37.5)	
HDMTX-based polychemotherapy	15 (39.4)	10 (62.5)	
**Frontline rituximab†**	33 (64.7)	9 (56.3)	0.566
**Response to initial therapy†**			0.042
CR/CRu	40 (78.4)	16 (100)	
PR	11 (21.6)	0 (0)	

‡ Median (range)

† Number of patients (%)

Abbreviations: CSF, cerebrospinal fluid; ECOG PS, Eastern Cooperative Oncology Group Performance Status; LDH, lactate dehydrogenase; IELSG, International Extranodal Lymphoma Study Group; MSKCC, Memorial Sloan Kettering Cancer Center; HDMTX, high-dose methotrexate; CR, complete response; CRu, unconfirmed complete response; PR, partial response.

Supplementary figure legends

Fig. S1 Kaplan–Meier curves for (A) overall survival and (B) progression-free survival in patients with primary nervous system lymphoma (PCNSL).

Fig. S2 **Kaplan–Meier survival curves in 18 patients with stable disease/progressive disease who responded to the subsequent salvage therapies, stratified by the adoption of consolidation treatment or not.** Patients with consolidation treatment had a trend of better overall survival (OS) (A) and progression-free survival (PFS) (B) than those without consolidation treatment. Notably, OS and PFS were calculated from the time of achieving a complete or partial response after salvage treatment.

Fig. S3 **Kaplan–Meier survival curves in patients with primary central nervous system lymphoma who responded to the induction chemotherapy, stratified by the types of induction chemotherapy and the adoption of consolidation/maintenance (cons./main.) treatment or not.** In the high-dose methotrexate (HDMTX)-monotherapy group (A), the patients with cons./main. treatment had a trend of better overall survival (OS) as compared to those without treatment. Similarly, in the HDMTX-based-polychemotherapy group (B), the patients being treated also had a trend of better OS as compared to the patients without cons./main. treatment. Notably, OS was calculated from

the time of achieving a complete or partial response after induction treatment.

Fig. S1

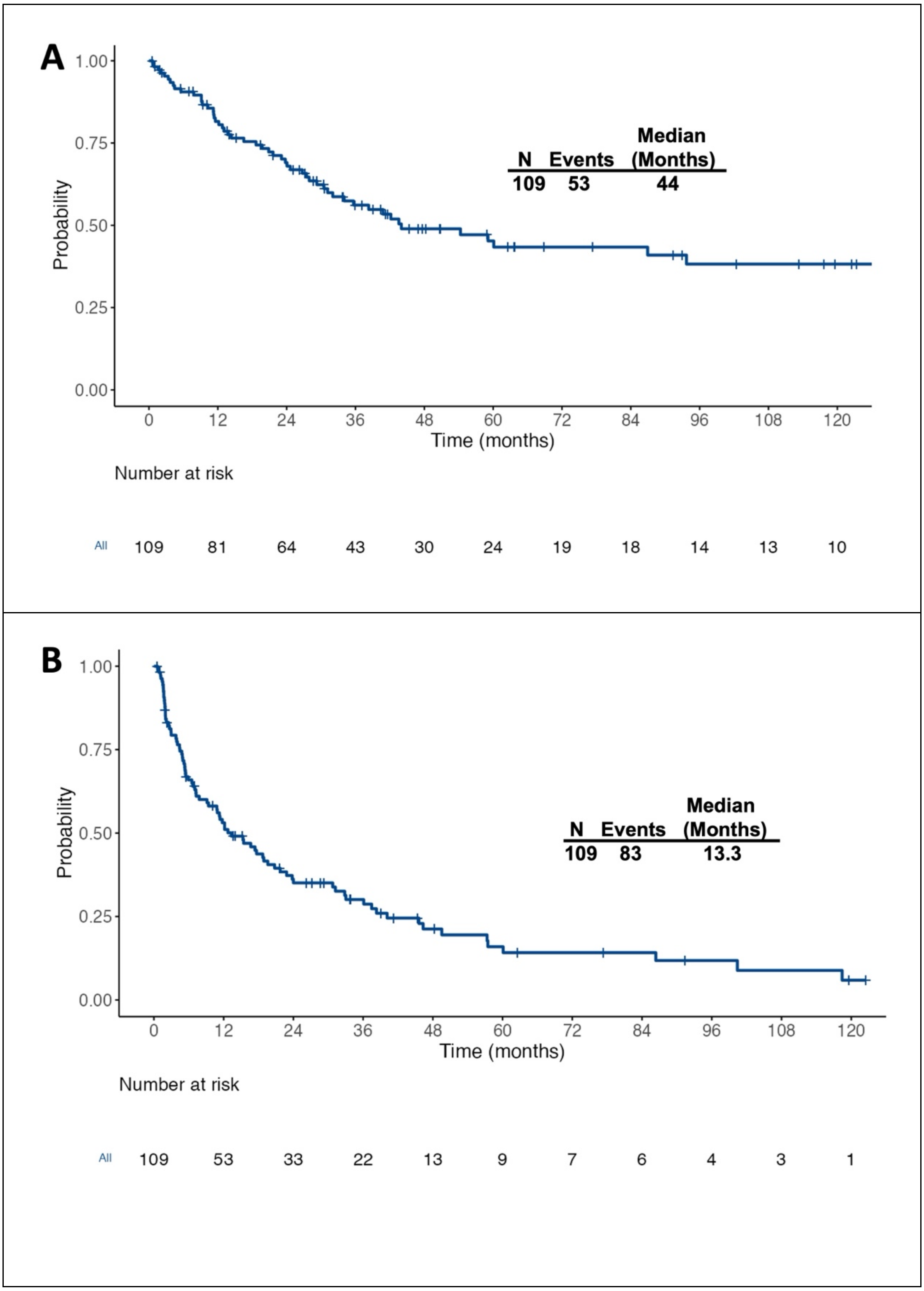
A
Probability
1.00
0.75
0.50
0.25
0.00
0 12 24 36 48 60 72 84 96 108 120
Time (months)
N Events Median (Months)
109 53 44
Number at risk
All 109 81 64 43 30 24 19 18 14 13 10
B
Probability
1.00
0.75
0.50
0.25
0.00
0 12 24 36 48 60 72 84 96 108 120
Time (months)
N Events Median (Months)
109 83 13.3
Number at risk
All 109 53 33 22 13 9 7 6 4 3 1

Fig. S2

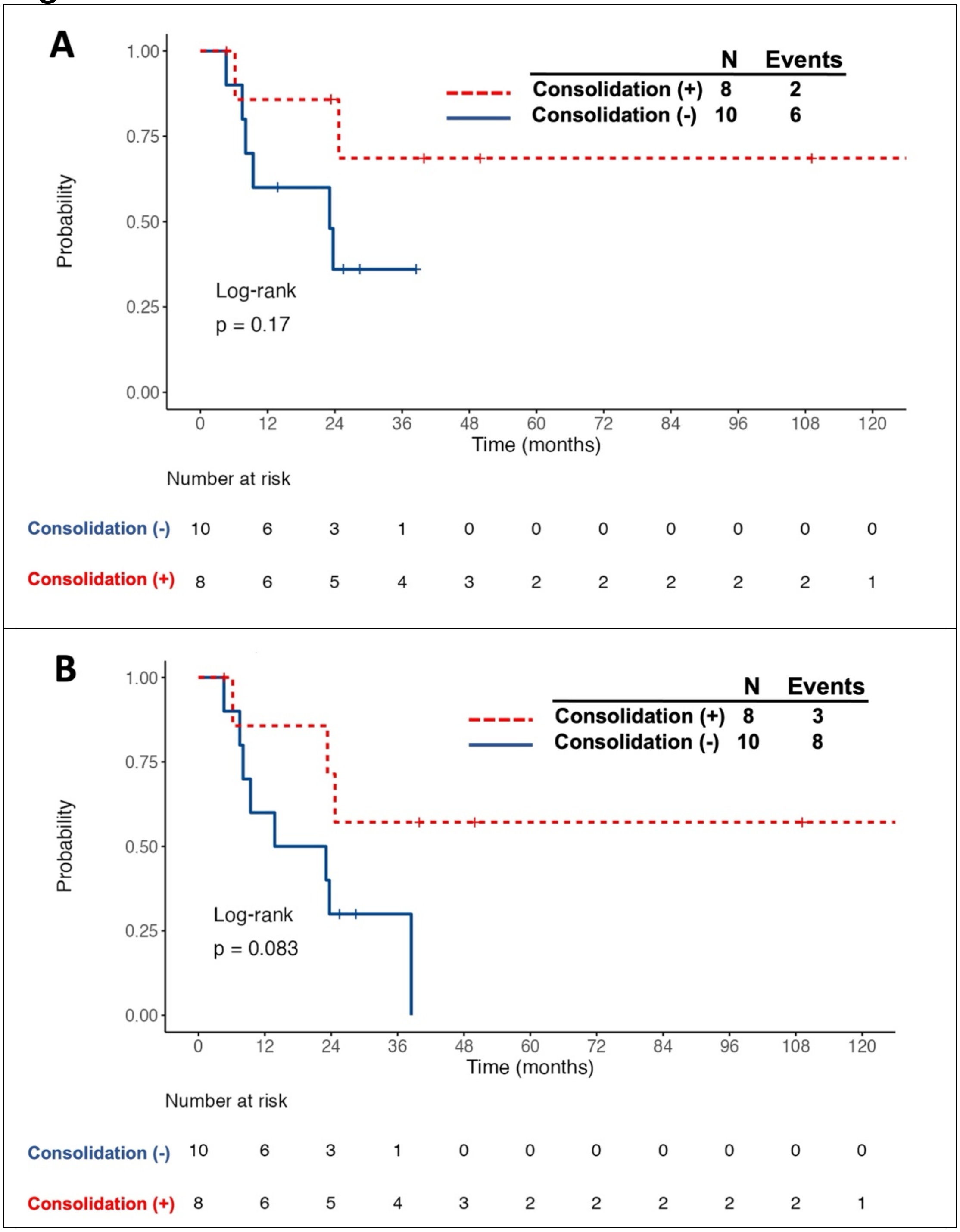
A
N Events
Consolidation (+) 8 2
Consolidation (-) 10 6
Probability
1.00
0.75
0.50
0.25
0.00
Log-rank
p = 0.17
0 12 24 36 48 60 72 84 96 108 120
Time (months)
Number at risk
Consolidation (-) 10 6 3 1 0 0 0 0 0 0 0
Consolidation (+) 8 6 5 4 3 2 2 2 2 2 1
B
N Events
Consolidation (+) 8 3
Consolidation (-) 10 8
Probability
1.00
0.75
0.50
0.25
0.00
Log-rank
p = 0.083
0 12 24 36 48 60 72 84 96 108 120
Time (months)
Number at risk
Consolidation (-) 10 6 3 1 0 0 0 0 0 0 0
Consolidation (+) 8 6 5 4 3 2 2 2 2 2 1

Fig. S3

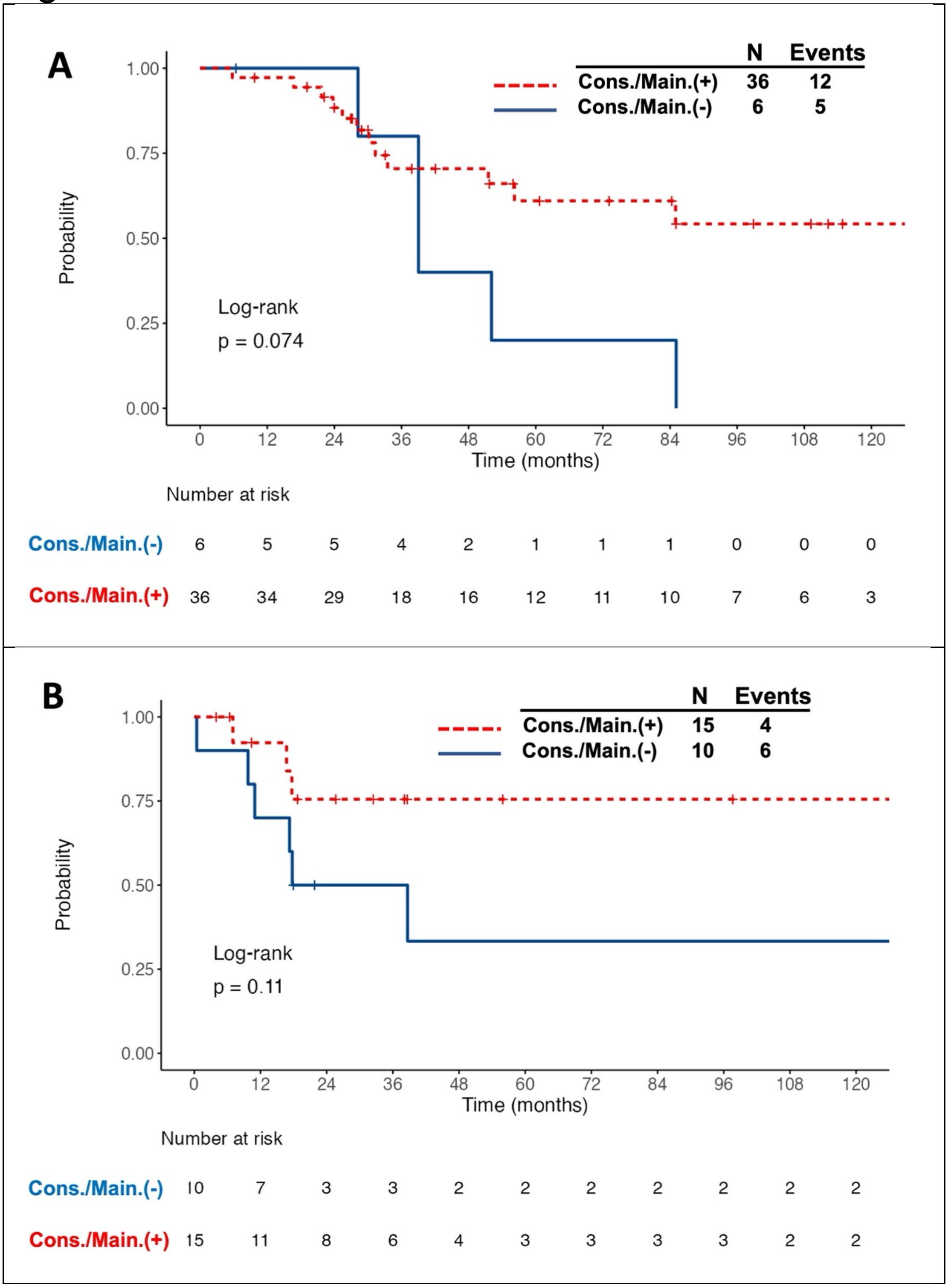
A
N Events
Cons./Main.(+) 36 12
Cons./Main.(-) 6 5
Probability
1.00
0.75
0.50
0.25
0.00
Log-rank
p = 0.074
0 12 24 36 48 60 72 84 96 108 120
Time (months)
Number at risk
Cons./Main.(-) 6 5 5 4 2 1 1 1 0 0 0
Cons./Main.(+) 36 34 29 18 16 12 11 10 7 6 3
B
N Events
Cons./Main.(+) 15 4
Cons./Main.(-) 10 6
Probability
1.00
0.75
0.50
0.25
0.00
Log-rank
p = 0.11
0 12 24 36 48 60 72 84 96 108 120
Time (months)
Number at risk
Cons./Main.(-) 10 7 3 3 2 2 2 2 2 2 2
Cons./Main.(+) 15 11 8 6 4 3 3 3 3 2 2
